# Lipidomic Signature of Pregnant and Postpartum Females by Longitudinal and Transversal Evaluation: Putative Biomarkers Determined by UHPLC-QTOF-ESI^+^-MS

**DOI:** 10.3390/metabo15010027

**Published:** 2025-01-08

**Authors:** Alexandra Traila, Marius Craina, Carmen Socaciu, Andreea Iulia Socaciu, Diana Nitusca, Catalin Marian

**Affiliations:** 1 Doctoral School, “Victor Babes” University of Medicine and Pharmacy Timisoara, Eftimie Murgu Square 2, 300041 Timisoara, Romania; alexandra.traila@umft.ro; 2 Obstetrics and Gynecology Clinic, Emergency Municipal Clinical Hospital, 300172 Timisoara, Romania; 3 Department of Obstetrics and Gynecology, “Victor Babes” University of Medicine and Pharmacy Timisoara, Eftimie Murgu Square 2, 300041 Timisoara, Romania; craina.marius@umft.ro; 4 Biodiatech, Research Center for Applied Biotechnology in Diagnosis and Molecular Therapy, 400478 Cluj-Napoca, Romania; carmen.socaciu@usamvcluj.ro; 5 Department of Occupational Health, Iuliu Hateganu University of Medicine and Pharmacy, Str. Victor Babes Nr. 8, 400347 Cluj-Napoca, Romania; 6 Department of Biochemistry and Pharmacology, Victor Babes University of Medicine and Pharmacy, Pta Eftimie Murgu Nr. 2, 300041 Timisoara, Romania; cmarian@umft.ro; 7 Center for Complex Networks Science, Victor Babes University of Medicine and Pharmacy, Pta Eftimie Murgu Nr. 2, 300041 Timisoara, Romania

**Keywords:** lipidomics, biomarkers, metabolomics, pregnancy, postpartum

## Abstract

Background: Pregnancy induces significant physiological and metabolic changes in the mother to support fetal growth and prepare for childbirth. These adaptations impact various systems, including immune tolerance, metabolism, and endocrine function. While metabolomics has been utilized to study pregnancy-related metabolic changes, comprehensive comparisons between pregnant and non-pregnant states, particularly using ultra-high-performance liquid chromatography coupled with mass spectrometry (UHPLC-MS), remain limited. Methods: This study aimed to explore the dynamic, longitudinal metabolic shifts during pregnancy by profiling plasma samples from 65 pregnant women across three time points (6–14 weeks, 14–22 weeks, and >24 weeks) and 42 postpartum women. Lipidomics was prioritized, and a solvent mixture was employed to enhance lipid extraction, using UHPLC-QTOF-ESI^+^-MS. Results: A total of 290 metabolites were identified and analyzed. Our results revealed significant metabolic differences between pregnant and postpartum women, with lipid molecules such as estrogen derivatives, fatty acids, and ceramides showing strong potential as biomarkers. Further biomarker analysis highlighted distinct metabolic signatures between early and late pregnancy stages, particularly in lipid metabolism (with AUC values > 0.8). Conclusions: These findings contribute to a deeper understanding of pregnancy-related metabolic changes and may offer insights into maternal and neonatal health outcomes.

## 1. Introduction

Pregnancy involves a plethora of profound adaptive physiological changes in the mother, which subsequently facilitate fetal growth and prepare her for childbirth. These changes could encompass immune tolerance of the placenta, metabolic adjustments to meet the increased nutrient and oxygen demands of the fetus, and significant endocrine shifts in response to pregnancy hormones like human chorionic gonadotropin (hCG). Therefore, these complex adaptations impact multiple systems, including maternal blood volume, cardiovascular function, kidney filtration, and immune tolerance, indicating that pregnancy is a rigorous “stress test” for maternal physiology [1,2,3].

In this regard, to explore these interconnected physiological changes at a system level, metabolomics—a tool for analyzing small molecules in blood and other biological samples—offers valuable insights into the various metabolic states during pregnancy [4,5]. Metabolomics has been previously used to differentiate between normal and complicated pregnancies, including conditions such as pre-eclampsia, preterm labor, and intrauterine growth restriction [6,7,8]. However, comprehensive comparisons between metabolic profiles in pregnant and non-pregnant states remain limited. Recent studies using nuclear magnetic resonance (NMR) have identified shifts in metabolites, such as branched-chain amino acids, cytokines, steroid hormones, and lipids between pregnant and non-pregnant women, but the more sensitive ultra-high-performance liquid chromatography coupled with mass spectrometry technique (UHPLC-MS), which can detect a broader range of metabolites, has not been extensively applied in this context [9,10].

Pregnancy is a period characterized by continuous and complex metabolic adaptations that support the growing fetus and prepare the maternal body for childbirth and lactation. Despite the crucial role of these metabolic changes, the dynamic, longitudinal shifts in specific metabolites throughout gestation remain insufficiently investigated. Existing studies that have tracked the plasma levels of metabolites—such as amino acids, fatty acids, and acylcarnitines—provide valuable insights but often rely on fixed sampling points and are constrained by limited sample sizes, reducing their ability to detect subtle or variable patterns across different stages of gestation and among diverse populations. For instance, Handelman et al. demonstrated that the plasma metabolome of women in early pregnancy differs significantly from that of non-pregnant women, emphasizing the substantial metabolic reprogramming that occurs at the onset of gestation [11]. Similarly, Ryckman et al. investigated how pregnancy-related changes in amino acid and acylcarnitine concentrations are modulated by maternal obesity, highlighting an additional layer of complexity [12]. Orczyk-Pawilowicz et al. extended this research by examining the metabolomics of maternal plasma and amniotic fluid, offering insights into the interactions between maternal and fetal compartments. While these studies have enriched our understanding, their focus on specific time points underscores the need for more comprehensive, longitudinal analyses to uncover the dynamic trajectory of metabolic changes throughout gestation.

In response to these gaps, our study utilized a diverse, longitudinal cohort of pregnant women where plasma sampling was performed at different time points (6–14 weeks, 14–22 weeks and >24 weeks) to map trajectories of metabolites throughout pregnancy. The levels of these metabolites were compared to those of postpartum females (1, 2, or >3 days following natural or cesarean birth).

This work mainly focused on lipidomics; therefore, a protocol using a mixture of solvents (methanol with acetonitrile and methyl tert-butyl ether) was applied to enhance the extraction of lipid components, since lipid compounds play a more significant role in the metabolism of pregnant women, particularly in hormonal synthesis [12,13,14,15,16,17]. Our research aimed to clarify natural metabolic shifts across pregnancy, which could illuminate underlying mechanisms in maternal and neonatal health outcomes.

## 2. Materials and Methods

### 2.1. Study Participants and Sample Collection

The present study included 107 subjects in total, out of which 65 were pregnant women, categorized as follows: 20 women in weeks 6–14, 17 women in weeks 14–22, and 28 women in >24 weeks of pregnancy. The control group included 42 postpartum females (23 had their blood collected at 1–2 days following natural (10) or cesarian (13) birth, and 19 women had their blood collected at more than 3 days following natural (7) or cesarian (12) birth).

Blood samples were collected at the Obstetrics and Gynecology Clinic of the Emergency Municipal Clinical Hospital, Timisoara, Romania. Each participant provided informed consent for the use of their biological samples. This study was conducted in accordance with the 1964 Declaration of Helsinki and its subsequent amendments and was approved by the Ethics Committee of the University of Medicine and Pharmacy “Victor Babes” Timisoara, Romania (approval code no. 28/01.09.2023, approval date 1 September 2023), and by the Ethics Committee of the Timis County Emergency Clinical Hospital, Timisoara, Romania (approval code no. 424/08.12.2023, approval date 12 December 2023). Venous blood samples were collected in EDTA-coated tubes, with plasma immediately separated by centrifugation (15 min at 2000× *g*) and stored at −80 °C for further analysis. Table 1 presents the clinical and demographic characteristics of the 107 participants included in this study.

### 2.2. Sample Processing

To 0.2 mL of plasma, 0.8 mL of a solvent mixture (methanol/acetonitrile/tert-butyl ether, 1:1:0.25) was added. The mixture was vortexed for 30 s and then stored at −20 °C for 24 h to allow protein precipitation. After thawing, the samples were centrifuged at 12,500× *g* for 10 min. The supernatant was collected, filtered through 0.2 µm PTFE filters, and transferred to autosampler vials for metabolomic analysis. QC samples were obtained by mixing 0.2 mL of plasma from each sample, carried out at the beginning and after every 10 samples to check the reproducibility of the LC-MS analysis.

### 2.3. UHPLC-QTOF-ESI^+^-MS Analysis

The UHPLC-MS analysis was conducted using a Bruker Daltonics MaXis Impact (Bruker GmbH, Bremen, Germany) system, which included a Thermo Scientific HPLC UltiMate 3000 setup featuring a Dionex Ultimate quaternary pump and ESI+-QTOF-MS detection. The analysis was performed on a C18 reverse-phase column (Kinetex, UPLC C18, 5 µm, 4.6 × 150 mm, Phenomenex, Torrance, CA, USA) at 25 °C with a flow rate of 0.8 mL/min. An injection volume of 25 microliters was used. The mobile phase consisted of a gradient of eluent A (water containing 0.1% formic acid) and eluent B (methanol/acetonitrile/isopropanol 1:1:1, containing 0.1% formic acid). The gradient profile was as follows: 70% A at min 0, 30% A at min 4, 0% A at min 7, 30% A at min 10, and returning to 70% A at min 13, followed by an additional 2 min with 70% A. The total run time was 15 min. All measurements were conducted in duplicate. If differences were found, a third run was conducted. Finally, the average matrix was considered.

The mass spectrometry parameters were configured for a mass range of 100 to 1000 Da. The nebulizing gas pressure was set to 2.8 bar, the drying gas flow was maintained at 12 L/min, and the drying gas temperature was set to 300 °C. Calibration with sodium formate was performed before each chromatographic run. Instrument control and data processing utilized specific software from Bruker Daltonics, including Chromeleon, TofControl 3.2, Hystar 3.2, and Data Analysis 4.2.

### 2.4. Statistical Analyses

Data processing was performed using Data Analysis 4.2. software. Initially, individual total ion chromatograms (TICs) were recorded and subsequently transformed into base peak chromatograms (BPCs). Compound spectra were obtained using the FMF (Find Molecular Features) function. The output table from the FMF matrix included the retention times, peak areas and intensities, and signal-to-noise (S/N) ratio for each component, along with their respective *m*/*z* values. Typically, the number of separated compounds ranged from 1100 to 1800 molecules. Molecules with retention times shorter than 1.6 min (corresponding to the void volume of the LC column) and peak intensities below 3000 were excluded, as were those with S/N values below 10.

**Figure 1:**
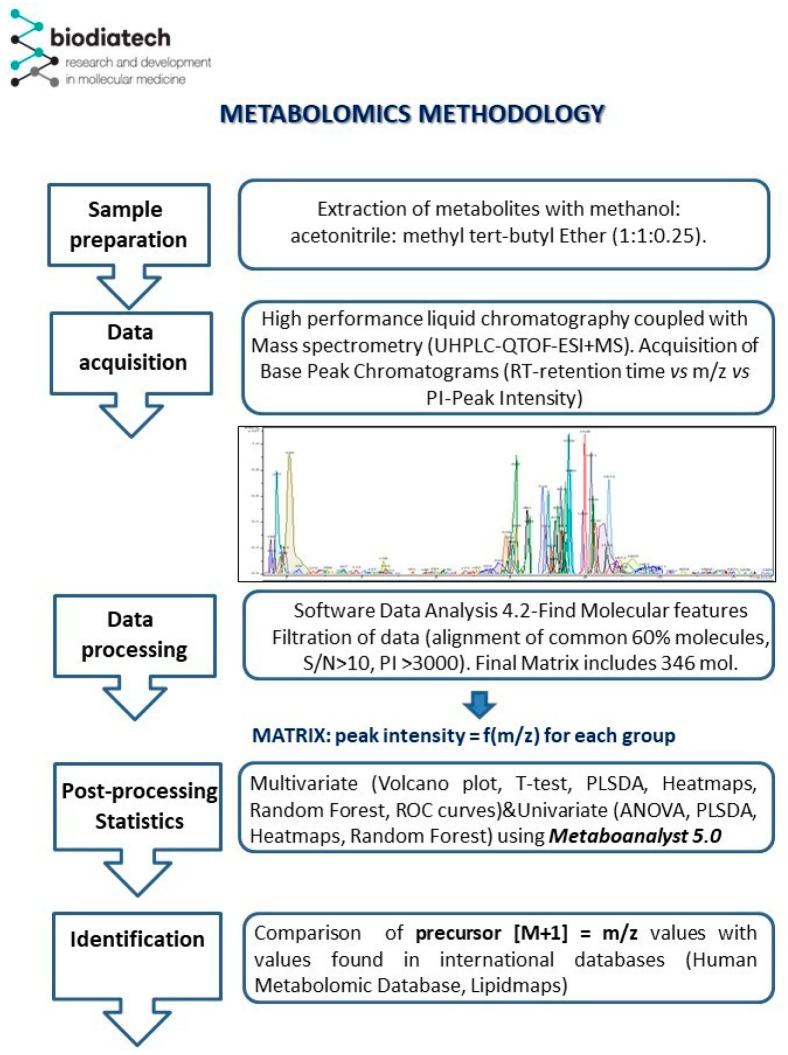
Metabolomics methodology (all steps applied in this study).

The *m*/*z* values of 60% of the common molecules identified in the samples were aligned using software available at https://www.bioinformatics.org/bioinfo-af-cnr/NEAPOLIS/ (accessed on the 4 November 2024), resulting in a matrix containing a number of 346 *m*/*z* values. The web tool Neapolis was applied for the alignment of signals from mass spectra and then submitted for statistical analysis as a .csv file, being uploaded to the online platform Metaboanalyst 5.0 (www.metaboanalyst.com, accessed on the 4 November 2024). An overview of the methodology applied in this study is illustrated in Figure 1.

## 3. Results

The demographic and clinical features of the subjects can be seen in Table 1. The mean age of pregnant women (all stages of pregnancy) was between 27.3 and 28.5 years old and matched with the control group of postpartum women (28.9). Nearly 60% of the control group consisted of postpartum females who opted for cesarean birth. More than half of the control group (which consisted of 13 women who opted for C-section and 10 who opted for natural birth) had their blood collected momentarily after birth (1–2 days postpartum).

Out of 346 *m*/*z* values, a number of 290 metabolites were identified, and they are listed in Supplementary Table S1. This table presents the *m*/*z* values for the precursor ions (adduct [M + H+]), which were identified as potential biomarkers through comparison with average isotopic mass and a mass tolerance of 0.05 Da based on data from the HMDB and LipidMaps databases (with corresponding IDs included). The metabolites, along with their putative names, were identified using these databases.

**Figure 2:**
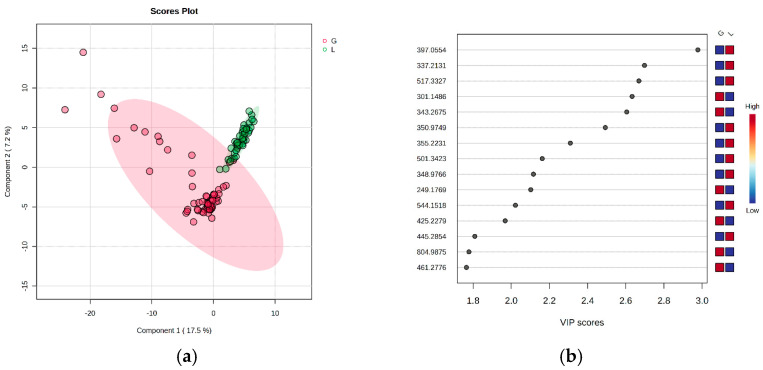
(**a**). Partial least squares discriminant analysis (PLSDA) plot showing the discrimination between groups. (**b**) PLSDA loadings plot, showing the VIP scores of the main 15 molecules selected as representative for the discrimination between groups (G = pregnant group; L = postpartum group).

According to the supervised Partial Least Squares Discriminatory Analysis (PLSDA) plot, the co-variance for the first five components was evaluated. The explained co-variance of the pregnant group vs. the control group was 24.7%, with the discrimination being significant (Figure 2a). From the PLSDA loadings plot, we selected the most relevant molecules that could be considered responsible for the discrimination. Figure 2b represents the *m*/*z* values of these molecules and the Variable of Importance in Projection (VIP) scores above 1 for the top 15 molecules, as well as their relative variation (higher or smaller and colored in red and blue, respectively).

According to the PLSDA plot, the pregnant group was less homogeneous than the control group, and this can be explained by the different timings of the pregnancy. The cross-validation algorithm when the first five components were considered indicated an accuracy of over 0.9, a maximal value of R2 = 0.75, and Q2 values around 0.7, suggesting good predictability for this model.

Using MetaboAnalyst 6.0 software, biomarker analysis incorporated the receiver operating characteristic (ROC) curve, particularly focusing on the area under the ROC curve (AUC) as an indicator of predictive accuracy. The ROC curve illustrated sensitivity versus specificity for each molecule, aiding in the identification of relevant biomarkers. Higher AUC values, approaching 1, indicated stronger predictive potential as biomarkers. Table 2 lists the *m*/*z* values, putative identifications, AUC values, *p*-values, and log_2_FC values for each identified molecule, along with their variations between the pregnant (all stages) and postpartum groups (natural and cesarean birth).

High AUC values above 0.75 indicate that 20 molecules may serve as potential biomarkers, with most belonging to various lipid classes. These findings suggest that lipid molecules—particularly estrogenic hormone derivatives, long-chain fatty acids, and ceramides—are promising potential biomarkers with strong prognostic value for differentiation.

**Figure 3:**
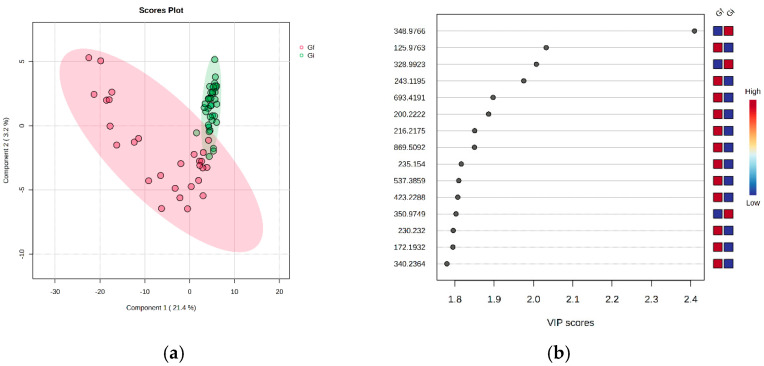
(**a**) PLSDA score plot showing discrimination between the Gi (<24 weeks) and Gf (>24 weeks) groups. (**b**) PLSDA loadings plot including the VIP scores of the top 15 molecules selected as representative for the discrimination between the Gi and Gf groups.

Next, we performed an untargeted multivariate analysis of pregnant groups Gi (<24 weeks) vs. Gf (>24 weeks) to detect potential differences in various pregnancy time points. By supervised PLSDA analysis, the co-variance for the first five components was evaluated. The explained co-variance of groups Gi and Gf was 24.8%, with the discrimination being significant (Figure 3a). Figure 3b represents the loadings graph showing the top 15 molecules (*m*/*z* values) and their corresponding VIP scores above 1 as a measure of discrimination between groups Gi and Gf.

According to the PLSDA plot, in this case, the Gi group was less homogeneous than the Gf group; this can be explained by the metabolic diversity of the Gi group, due perhaps to stronger metabolic modifications during the first period of pregnancy.

Biomarker analysis was performed as previously described. Table 3 shows the *m*/*z* value and putative identification, AUC value, *p*-value, and log_2_FC value for each molecule identified, as well as its variation in the Gi group vs. the Gf group.

In this case, AUC values exceeding 0.8 indicate that 20 molecules could be considered potential biomarkers, with these molecules spanning various lipid classes. This selection aligns well with complementary data from other algorithms, including Random Forest and VIP values.

Moreover, for the control group consisting 42 of females (who gave birth through C-section or natural birth), an additional statistical evaluation was conducted to assess the potential influence of blood collection timing on the metabolic profile. Each group was divided based on postpartum blood collection timing: N1 (natural birth, 10 females) and C1 (C-section, 13 females) were collected 1–2 days postpartum, while N2 (natural birth, 7 females) and C2 (C-section, 12 females) were collected more than 3 days postpartum. The co-variance for the first two components was 13.1%, the discrimination being nonsignificant according to the cross-validation algorithm. Higher levels of ceramides, triglycerides, and lysophosphatidylcholines were observed in subgroup C1 (C-section, blood collected after 1–2 days postpartum) compared to both C2 and N2 (C-section and natural birth, respectively, blood collected > 3 days following childbirth). Ceramides are known to play a key role in various physiological metabolic pathways. In this case, we hypothesized that their synthesis and function were particularly elevated during the early, postpartum period.

The initial nontargeted multivariate analysis, which provided an overview of potential biomarkers for distinguishing between female groups and subgroups, was followed by a more detailed univariate semi-targeted analysis. This subsequent analysis focused on specific metabolite classes that demonstrated discriminatory potential.

**Figure 4:**
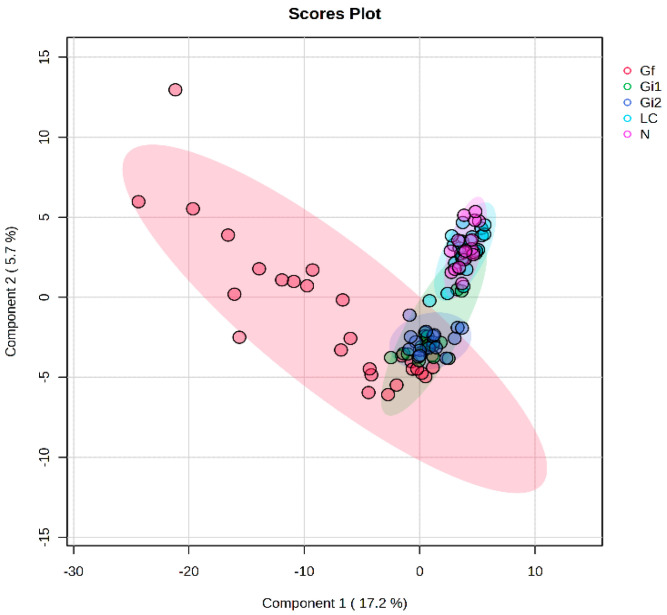
PLSDA score plot showing the discrimination between all five subgroups: 6–14 weeks of pregnancy, 14–22 weeks of pregnancy, >24 weeks of pregnancy, postpartum women with C-section, and natural birth.

The univariate analysis was conducted on five female subgroups as follows: Gi1, consisting of 20 pregnant women with a mean of 9.7 weeks of pregnancy; Gi2, consisting of 17 pregnant women with a mean of 18 weeks of pregnancy; Gf, consisting of 28 women with a mean of 34 weeks of pregnancy; N, consisting of 17 women who gave birth naturally; and C, consisting of 25 women who gave birth through C-section. A semi-targeted approach was applied to all 290 molecules belonging to different biochemical classes, especially derived from the lipid metabolism (phospholipids, free fatty acids, carnitine derivatives, neutral long-chain lipids, and steroid hormones), but also to polar molecules. For each molecular class, PLSDA score plots, loadings (VIP values), MDA-RF, and heatmaps were generated. The PLSDA score plot can be seen in Figure 4.

**Figure 5:**
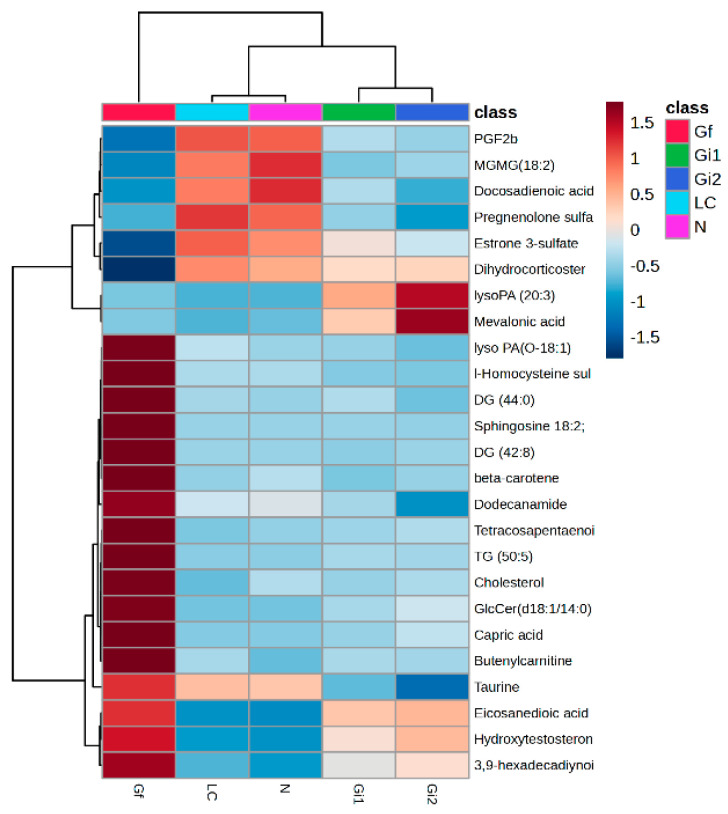
A heatmap for mean values for the five subgroups with the identification of molecules.

A heatmap applied for the mean values of each subgroup can be seen in Figure 5, including the identification with putative names for the most representative molecules.

**Figure 6:**
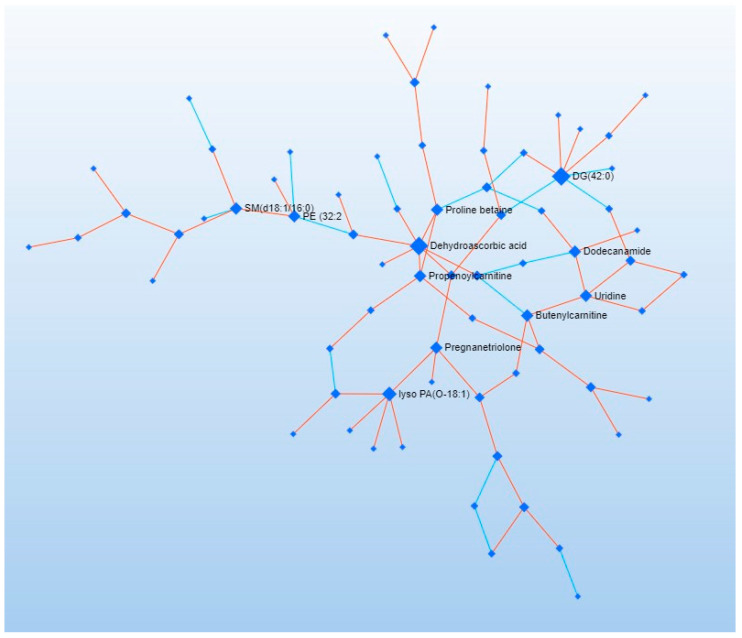
DSPC network of molecular relationships in the metabolic pathways (KEGG pathway database).

Additionally, a Debiased Sparse Partial Correlation (DSPC) network of molecular interactions within metabolic pathways was constructed based on the KEGG pathway database. The DSPC network comprises nodes (input metabolites) and edges representing associations, highlighting the top 20% of correlations by *p*-value ranking (Figure 6).

General observations, based on the algorithms and pathway analysis, include the following:

- Group homogeneity varied, with group Gf (>24 weeks of pregnancy) displaying a broader distribution of metabolic profiles, indicating lower homogeneity.
- When analyzing all 290 metabolites, group Gf showed higher levels compared to group Gi (<24 weeks of pregnancy), while no significant metabolic differences were observed between groups C and N.
- Among metabolite classes, hormones, long-chain fatty acids, lipids, and carnitines were key contributors to differentiating the pregnant groups Gi and Gf. The most pronounced metabolic changes were observed between these groups and were influenced by the pregnancy stage. No significant differences were detected between profiles for Gi1 (6–14 weeks) and Gi2 (14–22 weeks).

## 4. Discussion

The study presented here provides a comprehensive analysis of the various and complex metabolic changes occurring throughout pregnancy using UHPLC-QTOF-ESI^+^-MS. This study involved a total of 107 participants, including 65 pregnant women. These women were grouped based on their pregnancy stages: 20 in weeks 6–14, 17 in weeks 14–22, and 28 in weeks 24 and beyond. The control group consisted of 42 postpartum women, with 23 having their blood samples collected 1–2 days after delivery (10 following natural birth and 13 after cesarean section), while 19 women had their blood samples collected more than 3 days postpartum (7 following natural birth and 12 after cesarean section). Our investigation reveals distinct metabolic shifts across different stages of pregnancy and postpartum, with a particular emphasis on lipid metabolites. These findings align with previous studies showing that pregnancy induces substantial metabolic and hormonal adaptations to support fetal development, maternal homeostasis, and preparation for childbirth [18,19].

Pregnancy is a highly dynamic physiological state characterized by significant metabolic shifts, which are essential for fetal development and maternal adaptation. As observed in this study, lipid metabolism is one of the most profoundly affected areas. A large body of literature supports the idea that during pregnancy, there is an increased reliance on lipids as an energy source to meet the high metabolic demands of both the mother and fetus. Our results corroborate this by revealing significant changes in lipid metabolites, including fatty acids, ceramides, and estrogen derivatives, which are crucial for fetal development [20,21].

The comparison between pregnant and postpartum women revealed significant metabolic differences, which is consistent with earlier studies [22]. Our analysis also highlighted significant metabolic changes at different stages of pregnancy. Early pregnancy is typically characterized by increased insulin sensitivity and alterations in lipid metabolism to support the developing embryo. As pregnancy progresses, the maternal metabolic system shifts towards increased insulin resistance to ensure adequate nutrient supply for fetal growth [23]. In this study, we observed that the transition from early to late pregnancy was associated with increased levels of lipid metabolites, including fatty acids and ceramides, reflecting these well-documented physiological changes.

In any case, it should be emphasized that the categories of metabolites that differentiate pregnant women from postpartum women are primarily hormones. Additionally, by comparing the categories of molecules, it can be observed that the most significant discrimination occurs with hormones, and less so with phospholipids or neutral lipids, as suggested by the results of the present work. Estrogen derivatives, which increase during pregnancy, have been associated with the regulation of placental function, fetal development, and maternal metabolic adaptations [24,25]. The identification of specific lipid molecules (hormones included) as biomarkers may enhance our understanding of pregnancy-related metabolic changes and could offer a means of monitoring maternal and fetal health. Additionally, the observed alterations in ceramides and fatty acids could serve as early indicators of metabolic disorders, such as gestational diabetes and pre-eclampsia, which have been linked to abnormal lipid metabolism [26,27,28,29,30].

However, it should be noted that the differences are determined by multiple factors, including the patients’ genetic backgrounds, diets, and lifestyles, with the role of these factors being particularly important in pregnant women [31,32,33].

Therefore, while the findings from this study provide significant insights into metabolic adaptations during pregnancy, there are several limitations. First, the study design focused on a cohort of 65 pregnant and 42 postpartum women, which may not fully capture the diversity of metabolic changes in larger populations. Additionally, no information regarding the lifestyle background of the females included in this study (including diet and physical activity) was provided, which could greatly impact the level of lipid metabolites.

Differential metabolites observed during pregnancy can serve as crucial biomarkers for monitoring maternal and fetal health, predicting pregnancy complications, and tailoring personalized interventions. By identifying specific metabolites that deviate from normal trajectories, clinicians can intervene earlier to mitigate risks, improving outcomes for both the mother and the fetus. Moreover, the knowledge gained from studying metabolomic differences during pregnancy extends to improving precision medicine approaches. On a broader scale, longitudinal studies of the maternal metabolome can provide insights into fetal programming, where in utero exposures shape the long-term health of the child. Understanding how specific metabolomic shifts correlate with developmental outcomes could pave the way for preventive measures against chronic diseases, such as obesity, diabetes, and cardiovascular disorders, in later life.

However, for these benefits to be fully realized, research must overcome current limitations, such as small sample sizes, the lack of standardization in sampling protocols, and incomplete representation of diverse populations. Additionally, integrating metabolomic data with other omics approaches, including genomics and proteomics, can offer a more holistic view of pregnancy’s dynamic biological landscape. Future studies employing more extensive profiling of metabolites, along with longitudinal follow-ups, would provide a more holistic view of the metabolic changes occurring during pregnancy and postpartum recovery.

## 5. Conclusions

This study underscores the significant metabolic changes that occur during pregnancy, particularly in lipid metabolism, including steroid hormones. The identification of lipid metabolites such as estrogen derivatives, ceramides, phospholipids, and neutral lipids as potential biomarkers of pregnancy offers new avenues for understanding maternal and fetal health. Further exploration of the metabolic shifts across the different stages of pregnancy and postpartum, as well as their role in long-term maternal health, is essential for developing targeted interventions to improve pregnancy outcomes.

## Figures and Tables

**Table 1:** Demographic and clinical characteristics of pregnant and postpartum women.

Subjects	Mean Age	SD	Mean Weeks	SD
Pregnant < 24 weeks (*n* = 37)	28	4	14	5
Pregnant 6–14 weeks (*n* = 20)	28	5	10	2
Pregnant 14–22 weeks (*n* = 17)	29	3	18	3
Pregnant > 24 weeks (*n* = 28)	27	5	34	5
Postpartum all (*n* = 42)	29	6		
Postpartum C-section (*n* = 25)	29	6		
Postpartum natural birth (*n* = 17)	29	6		
Blood collection 1–2 days postpartum *n* = 23 (13 C-section,10 natural birth)	31	5		
Blood collection > 3 days postpartum, *n* = 19 (12 C-section, 7 natural birth)	26	7		

**Table 2:** The *m*/*z* values, area under the curve (AUC), *p*-values, and log_2_FC values for the putative biomarkers in blood plasma of pregnant vs. postpartum groups.

*m*/*z*	Identification	AUC	*p*-Value	Log_2_ FC	Relative Variation
301.1486	2-Methoxyestrone	0.861	2.35 × 10^−9^	1.961	POSTPARTUM > PREGNANT
343.2675	Eicosanedioic acid FA 20:1; O2	0.854	3.75 × 10^−9^	1.517	POSTPARTUM > PREGNANT
397.0554	Pregnenolone sulfate	0.843	3.81 × 10^−12^	−2.417	POSTPARTUM < PREGNANT
644.4431	GlcCer (d18:1/12:0)	0.834	2.54 × 10^−3^	0.004	POSTPARTUM > PREGNANT
350.9749	Estrone 3-sulfate	0.832	2.17 × 10^−8^	−1.134	POSTPARTUM < PREGNANT
820.5259	Cer(d18:1/35:0 (35OH))	0.832	4.31 × 10^−4^	0.197	POSTPARTUM > PREGNANT
701.4499	CerPE(d16:2 (4E,6E)/20:1 (11Z) (2OH))	0.817	2.01 × 10^−2^	0.416	POSTPARTUM > PREGNANT
337.2131	Docosadienoic acid acid FA 22:2	0.812	7.87 × 10^−10^	−1.672	POSTPARTUM < PREGNANT
356.3396	N-palmitoyl valine	0.812	1.31 × 10^−2^	1.349	POSTPARTUM > PREGNANT
246.1301	Valeroylcarnitine	0.810	3.22 × 10^−1^	−1.132	POSTPARTUM < PREGNANT
425.2279	Alpha-Tocotrienol	0.808	1.78 × 10^−5^	1.342	POSTPARTUM > PREGNANT
461.2776	LysoPA 20:3	0.805	1.37 × 10^−4^	1.117	POSTPARTUM > PREGNANT
501.3423	Palmitoleyl linolenate	0.792	1.94 × 10^−6^	−1.600	POSTPARTUM < PREGNANT
249.1769	3,9-hexadecadiynoic acid FA 16:4	0.791	3.97 × 10^−6^	0.172	POSTPARTUM > PREGNANT
544.1518	LysoPC (20:4)	0.788	9.93 × 10^−6^	−3.327	POSTPARTUM < PREGNANT
355.2231	PGF2β	0.787	2.95 × 10^−7^	−1.129	POSTPARTUM < PREGNANT
348.9766	Dihydrocorticosterone	0.787	3.37 × 10^−6^	−1.009	POSTPARTUM < PREGNANT
672.4711	GlcCer (d18:1/14:0)	0.782	1.85 × 10^−3^	−0.031	POSTPARTUM < PREGNANT
150.0856	Methionine	0.780	4.32 × 10^−4^	1.071	POSTPARTUM > PREGNANT
776.5121	1-O-palmitoyl-Cer (d18:1/16:0)	0.774	3.24 × 10^−3^	−0.055	POSTPARTUM < PREGNANT

Abbreviations: AUC = area under the curve; FC = fold change; FA = fatty acid; Cer = ceramide; PC = phosphatidylcholine, PA = phosphatidic acid; PGF = prostaglandin F.

**Table 3:** The *m*/*z* values and identification of molecules considered to be putative biomarkers, the area under the curve (AUC), *p*-values, and log_2_FC values, showing the relative variation between the Gi and Gf groups.

*m*/*z*	Identification	AUC	*p*-Value	Log_2_ FC	Relative Variation
693.4191	DG (42:8)	0.922	8.41 × 10^−7^	−0.217	Gi < Gf
348.9766	Dihydrocorticosterone	0.902	1.67 × 10^−11^	1.991	Gi > Gf
125.9763	Taurine	0.877	4.25 × 10^−7^	−0.770	Gi < Gf
810.5288	Cer(d18:1/35:0 (35OH))	0.874	1.01 × 10^−2^	−1.973	Gi < Gf
172.1932	L-Homocysteine sulfate	0.865	1.13 × 10^−5^	−0.638	Gi < Gf
537.3859	Beta-carotene	0.854	2.46 × 10^−6^	−2.140	Gi < Gf
216.2175	Propenoylcarnitine	0.846	4.42 × 10^−5^	0.398	Gi > Gf
298.3268	Sphingosine 18:2	0.841	4.58 × 10^−5^	−2.636	Gi < Gf
679.4567	DG (40:1)	0.840	6.57 × 10^−4^	−0.437	Gi < Gf
328.9923	Phenylalanyltyrosine	0.839	3.54 × 10^−8^	1.713	Gi > Gf
235.1540	5-Methoxytryptophan	0.832	1.59 × 10^−5^	0.332	Gi > Gf
149.0686	Mevalonic acid	0.831	7.98 × 10^−3^	−0.821	Gi < Gf
387.2326	Cholesterol	0.828	7.61 × 10^−6^	−0.199	Gi < Gf
825.4906	TG (50:5)	0.825	5.20 × 10^−5^	−0.213	Gi < Gf
173.1407	Capric acid	0.823	4.58 × 10^−5^	0.180	Gi < Gf
230.2320	Butenoylcarnitine	0.820	1.03 × 10^−5^	−0.808	Gi < Gf
577.3524	DG (33:3)	0.814	6.58 × 10^−5^	−0.683	Gi < Gf
243.1195	Thymidine	0.806	2.30 × 10^−6^	0.187	Gi > Gf
350.9749	Estrone 3-sulfate	0.803	2.21 × 10^−6^	1.578	Gi > Gf
427.2616	N-stearoyl arginine	0.803	1.91 × 10^−4^	0.202	Gi > Gf

Abbreviations: AUC = area under the curve; FC = fold change; DG = diglyceride; Cer = ceramide; TG = triglyceride.

## Data Availability

All data are available in this manuscript.

## Supplementary Materials

The following supporting information can be downloaded at https://www.mdpi.com/article/10.3390/metabo15010027/s1, Table S1: Molecules (*n* = 290) identified in plasma from a total of 346 molecules selected as common ones in all groups. Their identification was conducted using the HMDB and LipidMaps databases. IDs are included.
