# Supplementary material for: Lipidomic Signature of Pregnant and Postpartum Females by Longitudinal and Transversal Evaluation: Putative Biomarkers Determined by UHPLC-QTOF-ESI+-MS
Source: Metabolites. 2025 Jan 8;15(1):27. doi: 10.3390/metabo15010027 (PMC11768031; doi:10.3390/metabo15010027)
Supplement: Supplementary file 1 [file metabolites-15-00027-s001.zip › metabolites-3379742-supplementary.pdf]

Supplementary Table S1. Putative molecules (n=290) identified in plasma from a total of 346 molecules selected as common ones in all groups. Their putative identification was made using HMDB and LipidMaps data bases., ID being included.

m/z	Common molecules	ID
104.0982	Choline	HMDB0000097
109.0923	Phenylenediamine	HMDB0003119
111.1080	Pyrocatechol	HMDB0000957
115.1024	Dihydrouracil	HMDB0000076
116.0728	Proline betaine	HMDB0004827
120.0709	L-Threonine	HMDB0000167
123.0358	Erthrithol	HMDB0002994
125.9763	Taurine	HMDB0000251
129.1175	Dihydrothymine	HMDB0000079
132.0877	Leucine	HMDB0000687
146.0436	3-oxo-5-amino-hexanoic acid	LMFA01060173
146.0436	Spermidine	HMDB0001257
149.0686	Mevalonic acid	LMFA01050352
150.0856	Methionine	HMDB0000696
158.1433	Tiglylglycine	HMDB0000959
160.0498	Isovalerylglycine	HMDB0000678
162.0382	L-Carnitine	HMDB0000062
162.1003	Aminoadipic acid	HMDB0000510
163.0627	2-Hydroxyadipic acid	HMDB0000321
165.0977	Phenylpyruvic acid	HMDB0000205
166.0735	Phenylalanine	HMDB0000159
167.0844	Phenyllactic acid	HMDB0000779
172.1932	L-Homocysteine sulfate	HMDB0002238
173.1407	Capric acid	HMDB0000511
174.9915	Dehydroascorbic acid	HMDB0001264
175.1069	L-Arginine	HMDB0000517
177.0421	Serotonin	HMDB0000259
185.1014	Phosphorylcholine	HMDB0001565
188.0846	N1-Acetylspermidine	HMDB0001276
195.1225	3-O-Methyl-d-glucose	HMDB0245931
200.2222	Dodecanamide	HMDB0251566
202.2024	Cysteine-S-sulfate	HMDB0000731
203.0382	Sebacic acid	HMDB0000792
205.0771	Trytptophan	HMDB0000929
212.2229	Phosphocreatine	HMDB0001511

213.1329	Acetyl hydroxytryptamine	HMDB0001238
214.2374	Indoxyl sulfate	HMDB0000682
216.2175	Propenoylcarnitine	HMDB0013124
217.1366	3-Hydroxydodecanoic acid	HMDB0000387
223.1548	L-Cystathionine	HMDB0000099
227.1598	Myristoleic acid	HMDB0002000
228.2531	Deoxycytidine	HMDB0000014
229.1275	Traumatic acid	HMDB0000933
230.2320	Butenoylcarnitine	HMDB0013126
235.1540	5-Methoxytryptophan	HMDB0002339
239.1451	Glycyltyrosine	HMDB0028853
242.2675	Tetrahydrobiopterin	HMDB0000027
243.1195	Thymidine	HMDB0000273
245.0633	Uridine	HMDB0000296
246.1301	Valeroylcarnitine	HMDB0013128
249.1769	3,9-hexadecadiynoic acid C 16:4	LMFA01030491
250.9874	gamma-Glutamylcysteine	HMDB0001049
252.9843	Deoxyadenosine	HMDB0000101
256.2815	Palmitamide	LMFA08010009
258.2619	Glycerophosphocholine	HMDB0000086
261.1231	Androstane 19:0	LMST02020056
263.2207	11-Phenylundecanoic acid C 17:4	LMFA01140028
265.1144	Phenylacetylglutamine	HMDB0006344
267.2526	2,3-Diphosphoglyceric acid	HMDB0001294
269.1918	DL-Homocystine	HMDB0000575
271.1779	Estrone	LMST02010004
275.2224	Alfa-androstenol	LMST02020008
277.2000	Stearidonic acid C 18:4	LMFA01030357
279.1452	Estrane-3,17-diol ST 18:0;O2	LMST02010019
279.2151	Alpha-Linolenic acid C 18:3	LMFA01030152
281.1623	Linoleic acid C C18:2	LMFA01030120
284.3147	Stearamide	LMFA08010003
287.1453	Dehydro testosteron	LMST02020018
290.2672	O-adipoylcarnitine	LMFA07070087
293.2144	13S-HODTA-C 18:4;O	LMFA02000373
295.2183	13-HOTE - C 18:3;O	LMFA02000029
297.2244	13-HODE- C 18:2;O	LMFA02000035
298.3293	Sphingosine 18:2; O2	LMSP01080010
301.1486	2-Methoxyestrone	LMST02010033
304.8507	Hydroxy testosterone	LMST02020143
305.2766	Arachidonic acid C 20:4	LMFA01030001

307.1797	Eicosatrienoic acid C 20:3	LMFA01030157
309.1904	Eicosadienoic acid C 20:2	LMFA01030130
311.2207	Eicosenoic acid C 20:1	LMFA01030082
313.2276	Arachidic acid C20:0	LMFA01010020
315.0558	12,13-Dihydroxyoleic acid	LMFA02000302
315.1930	Progesterone	LMST02030159
317.1870	Pregnenolone	LMST02030088
318.2966	Phytosphingosine	LMSP01030001
319.2634	9-hydroxy-Eicosapentaenoic acid C 20:5;O	LMFA01030717
321.2715	Pregnanediol	LMST02030264
325.2515	Auricolic acid C 20:2;O	LMFA01050431
326.9949	14-hydroxy-11Z-eicosenoic acid	LMFA01050117
328.9923	Phenylalanyltyrosine	HMDB0029007
331.0373	17alpha-hydroxyprogesterone	LMST02030161
333.0428	21-hydroxypregnenolone	LMST02030167
335.2192	PGF2a	HMDB0001139
335.3243	PGA2	HMDB0002752
336.2934	Docosatrienoic acid C22:3	LMFA04000088
337.2131	Docosadienoic acid acid C 22:2	LMFA01170128
338.3235	16Z-docosenoic acid C 22:1	LMFA01031302
340.2364	Dodecadienoylcarnitine	LMFA07070124
340.3719	Oleoyl glycine	LMFA08020082
341.2452	Behenic acid C 22:0	LMFA01010022
342.3573	Dodecenoylcarnitine	LMFA07070115
343.2675	Eicosanedioic acid C 20:1; O2	LMFA01170035
343.3372	N,N,N-trimethyl-sphingosine	LMSP01080057
347.2954	Corticosterone	LMST02030186
348.9766	Dihydrocorticosterone	LMST02030280
350.9749	Estrone 3-sulfate	LMST02010043
351.2296	Pregnanetriolone	LMST02030290
354.3889	N-palmitoyl proline	LMFA08020117
355.2231	PGF2b	LMFA03010025
355.3568	10-oxo-docosanoic acid C22:1;O	LMFA01060139
356.3396	N-palmitoyl valine	LMFA08020120
357.2364	Tetracosahexaenoic acid C 24:6	LMFA01030804
359.2666	Tetracosapentaenoic acid C24:5	LMFA01030820
360.3396	2-Hydroxylauroylcarnitine	HMDB0013164
363.1979	Cortisol	LMST02030001
363.2887	Tetracosatrienoic acid C24:3	LMFA01031049
365.2250	Tetrahydroaldosterone	LMST02030275
368.4019	N-oleoyl GABA	HMDB0062335

369.2684	DHEAS	HMDB0001032
369.3512	Lignoceric acid C 24:0	LMFA01010024
380.3145	C18-Sphingosine 1-phosphate	HMDB0000277
383.1760	LysoPA (14:0)	HMDB0062321
387.2326	Cholesterol	HMDB0000067
391.3171	Ketodeoxycholic acid	HMDB0000328
395.2218	LysoPA(P-16:0)	HMDB0011154
397.0554	Pregnenolone sulfate	HMDB0000774
397.1954	Cerotic acid C26:0	HMDB0002356
399.2780	N-Palmitoyltryptamine	HMDB0040815
400.3535	Palmitoylcarnitine	HMDB0000222
401.2561	5,6-trans-25-Hydroxyvitamin D2	HMDB0006721
405.2407	Cortisol 21-acetate	LMST02030093
406.0536	12-HETE-GABA	LMFA08020147
407.3119	7-Ketodeoxycholic acid	LMST04010184
408.0499	N-linolenoyl glutamic acid	LMFA08020214
409.2234	LysoPA (16:1)	LMGP10050016
413.2668	7-Hydroxypregnenolone sulfate	LMST05020021
419.2596	7,27-dihydroxycholesterol	LMST04030178
423.2288	Lyso PA(O-18:1)	LMGP10060006
424.3364	O-linoleoylcarnitine CAR 18:2	LMFA07070092
425.2279	Alpha-Tocotrienol	LMPR02020054
427.2616	N-stearoyl arginine	LMFA08020136
432.2662	N-stearoyl phenylalanine	LMFA08020093
434.2642	N-arachidonoyl glutamic acid	LMFA08020086
437.2519	LysoPA (18:1)	LMGP10050008
439.2187	LysoPA (18:0)	LMGP10050005
442.3765	3-hydroxyoctadecenoylcarnitine CAR 18:1;O	LMFA07070025
443.3066	Cortisol 21- sulfate	LMST05020020
445.2854	Triacontatetraenoic acid C 30:4	LMFA01030826
449.2644	Fatty acid, C 30:2	LMFA01020366
452.5016	Eicosadienoylcarnitine CAR 20:2	LMFA07070011
452.6968	LysoPE (16:1)	LMGP02050010
453.3173	Myristyl palmitate	LMFA07010002
456.2225	Arachidyl carnitine	LMFA07070052
459.2976	LysoPA (20:4)	LMGP10050013
461.2776	LysoPA (20:3)	LMGP10050028
465.3666	Testosterone glucuronide	LMST05010012
468.3558	LysoPC 14:0	LMGP01050012
470.2415	LysoPS (14:0)	LMGP03050009
484.3541	O-behenoylcarnitine Car 22:0	LMFA07070089

487.3129	LysoPA (22:4)	LMGP10050020
491.2613	LysoPA (22:2)	LMGP10050030
494.5405	LysoPC(16:1)	LMGP01050021
496.3739	LysoPC(16:0)	LMGP01050018
501.3423	Palmitoleyl linolenate	LMFA07010121
511.3624	LysoPG (18:1)	LMGP04050006
512.3758	Cer(d16:0/16:0)	LMSP02020068
517.3327	MGMG(18:2)	LMGL04010010
522.5693	LysoPC (18:1)	LMGP01050029
524.3359	Cer(d18:2(4E,8E)/14:0(2OH))	LMSP02010216
528.2329	LysoPE (22:5)	LMGP02050058
528.3756	Cer(t18:0/14:0)	LMSP02030051
531.3522	Linolenyl stearate	LMFA07010148
533.3248	Linoleyl stearate	LMFA07010152
537.2974	Stearyl stearate	LMFA07010054
537.3859	beta-carotene	LMPR01070001
540.4093	Cer(d18:0/16:0)	LMSP02020001
544.1518	LysoPC (20:4)	LMGP01050048
550.5964	LysoPC(20:1)	LMGP01050047
556.4014	Cer(t18:1(6OH)/14:0(2OH))	LMSP02010195
560.1602	LysoPC (20:4;O)	LMGP01050147
561.3546	Linoleyl arachidate	LMFA07010164
562.2100	Cer(d18:2/18:1)	LMSP02010025
566.2869	Cer(d18:1/18:0)	LMSP02010006
568.2966	Cer(d18:0/18:0)	LMSP02020008
571.5822	LysoPI (16:1)	LMGP06050009
572.3939	LysoPC(22:4)	LMGP01050124
575.3746	DG(33:4)	LMGL02010368
577.3524	DG(33:3)	LMGL02010019
581.3191	DG(33:1)	LMGL02010013
583.2260	DG(33:0)	LMGL02010012
584.4274	Cer(d18:0/18:0(2OH))	LMSP02020030
589.3836	DG(34:4)	LMGL02010028
595.3426	DG(34:1)	LMGL02010004
597.4329	DG(34:0)	LMGL02010003
600.4213	Cer(t18:0/18:0(2OH))	LMSP02030016
605.3796	CerPE(d14:1/16:0)	LMSP03020002
609.2967	DG(35:1)	LMGL02010029
610.5348	Cer(t18:1(6OH)/20:0)	LMSP02010144
611.3233	DG(35:0)	LMGL02010025
619.4279	CerPE(d14:2/16:0(2OH))	LMSP03020066

625.3417	DG(36:0)	LMGL02010002
628.4495	Cer(t18:0/20:0(2OH))	LMSP02030017
629.0961	CerPE(d14:2/18:1))	LMSP03020031
631.3101	CerPE(d16:2/16:0)	LMSP03020047
633.4051	CerPE(d16:1/16:0)	LMSP03020019
634.4049	CerP(d18:1/16:0[2OH])	LMSP02050015
636.5553	Cer(d16:2/24:0(2OH))	LMSP02010093
638.5607	Cer(t18:1(6OH)/22:0)	LMSP02010142
639.3641	PA(32:5)	LMGP10010061
641.4382	DG (38:6)	LMGL02010130
644.4431	GlcCer(d18:1/12:0)	LMSP0501AA01
645.0653	CerPE(d15:2/18:0)	LMSP03020043
645.5167	N-palmitoyl-D-sphingosyl-1-(2-aminoethyl)phosphonate	LMSP04000002
649.3946	PA(16:0/16:0)	LMGP10010012
650.2559	Cer(d18:0/24:1(15Z))	LMSP02020011
660.4391	GlcAbeta-Cer(d16:0/14:0)	LMSP06030003
661.0296	CerPE(d16:1/18:0)	LMSP03020020
663.4148	SM(d18:0/13:0)	LMSP03010033
666.5878	Cer(d18:1/24:0(3OH))	LMSP02010204
669.3663	DG (40:6)	LMGL02010186
672.4711	GlcCer(d18:1/14:0)	LMSP0501AA26
675.4910	CerPE(d16:1/18:1)(2OH))	LMSP03020081
677.4284	CerPE(d16:1/18:0(2OH))	LMSP03020080
679.4567	DG (40:1)	LMGL02010128
685.3790	CerPE(d16:2/20:1)	LMSP03020051
688.4722	PE (32:2	LMGP02010108
689.6877	DG (41:3)	LMGL02010212
693.4191	DG (42:8)	LMGL02010260
694.3539	Cer(d18:1/26:0(3OH))	LMSP02010205
696.3574	Cer(t18:1(6OH)/25:0(2OH))	LMSP02010184
701.4499	CerPE(d16:2/20:1(2OH))	LMSP03020090
703.5173	SM(d18:1/16:0)	LMSP03010003
707.4405	CerPE(d18:0(17Me)/16:0(3OH,15Me))	LMSP03020095
709.4068	DG(42:0)	LMGL02010200
713.3910	DG (44:12)	LMGL02010306
713.4949	CerPE(d14:2/24:1(15Z))	LMSP03020037
716.4925	PC(P-16:0/16:1))	LMGP01030026
717.4072	CerPE(d16:1/22:0)	LMSP03020024
721.4475	DG (44:8)	LMGL02010296
723.4839	DG (44:7)	LMGL02010292
725.4988	DG (44:6)	LMGL02010282

727.4040	SM(d18:2/18:1)	LMSP03010047
729.5143	SM(d18:1/18:1)	LMSP03010029
730.4820	GlcCer(d18:0/18:0)	LMSP0501AA19
732.4944	GlcCer(t18:1/16:0(2OH))	LMSP05010042
737.4851	DG (44:0)	LMGL02010258
742.5059	GlcCer(d18:2/18:0(2OH))	LMSP05010050
744.5179	GlcCer(d18:1/18:0(2OH[R]))	LMSP05010060
752.4681	GlcCer(d18:2/20:1)	LMSP0501AA35
753.4267	MGDG (34:3)	LMGL05010056
754.4749	GlcCer(d18:2)(9Me)/18:1(2OH))	LMSP0501AA82
756.4931	GlcCer(d18:2(9Me)/18:0(2OH))	LMSP0501AA83
757.4086	CerPE(d16:2/24:1(2OH))	LMSP03020094
758.5085	GlcCer(d16:1(15Me)/20:0(2OH)	LMSP05010183
760.5253	Cer(d18:2/32:0)	LMSP02010178
765.4722	PS(18:0/16:0)	LMGP03010888
766.5104	Cer(t18:1(6OH)/30:0(2OH))	LMSP02010183
768.5206	Cer(t18:0/30:0(30OH))	LMSP02030042
776.5121	1-O-palmitoyl-Cer(d18:1/16:0)	LMSP02040003
780.4898	Cer(t18:0/32:0)	LMSP02030063
782.5064	GlcCer(d18:2/22:0)	LMSP0501AA37
784.5220	GlcCer(d18:1/22:0)	LMSP0501AA07
786.5326	GlcCer(d18:0/22:0)	LMSP0501AA21
788.5420	GlcCer(t18:1/20:0(2OH))	LMSP05010044
790.5100	GlcCer(t15:0(14Me)/22:0(2OH))	LMSP05010084
794.5335	Cer(t18:1(6OH)/32:0(32OH))	LMSP02010115
796.4637	Cer(t18:0/32:0(32OH))	LMSP02030044
798.4773	PI-Cer(d18:0/16:0(2OH))	LMSP03030030
801.4326	SM(d18:1/23:0)	LMSP03010078
804.5005	1-O-palmitoyl-Cer(d18:1/18:0)	LMSP02040011
806.5042	Cer(d18:1/34:0(34OH))	LMSP02010111
808.5184	PI-Cer(d20:1/16:0)	LMSP03030154
810.5288	Acetyl CoA	HMDB0001206
816.5513	GlcCer(t18:1/22:0(2OH))	LMSP05010045
818.5559	GlcCer(t16:0(15Me)/23:0(2OH))	LMSP05010078
820.5259	Cer(d18:1/35:0(35OH))	LMSP02010112
825.4906	TG (50:5)	LMGL03010056
828.4859	GlcCer(d18:1/24:0(2OH[R]))	LMSP05010063
832.5163	1-O-stearoyl-Cer(d18:1/18:0)	LMSP02040013
834.5313	Cer(d18:1/36:0(36OH))	LMSP02010113
840.5496	GlcCer(d18:1/26:0)	LMSP0501AA11
841.4642	SM(d18:1/26:1(17Z))	LMSP03010009

848.5609	PE (44:6)	LMGP02011100
853.5174	TG 52:5	LMGL03010140
858.3987	GlcCer(d18:1/25:0(2OH[R]))	LMSP05010046
864.5569	PI-Cer(d18:1/22:0)	LMSP03030001
869.5092	Glc-cholesterol 22:1	LMST01010380
892.5842	LacCer(d18:0/18:0)	LMSP0501AB15
908.5772	(3'-sulfo)Galbeta-Cer(d18:1/24:0(2OH))	LMSP06020014
957.5535	TG 60:9	LMGL03011621
991.5927	GM4(d18:1/16:0)	LMSP0601AA01
